# A growing trend of females and dermatologists among top medical graduates in 30 years

**DOI:** 10.1186/s12909-020-02028-1

**Published:** 2020-04-15

**Authors:** Yung-Wei Chang, Chih-Hung Lee

**Affiliations:** Department of Dermatology, Kaohsiung Chang Gung Memorial Hospital and Chang Gung University College of Medicine, 123 Dapi Rd, 83301 Kaohsiung, Taiwan

**Keywords:** Dermatologist, Female, Policy, Top medical graduate, Taiwan

## Abstract

**Background:**

Career outcomes of top medical graduates (TMG) are seldom studied. The Tsungming Tu Foundation (TTF) has awarded the number one graduate from each medical school in Taiwan since 1981. We aimed to study whether TMG differ from all medical graduates (AMG) in gender, specialty, and geographic regions in the last 30 years.

**Methods:**

Overall, 322 TMG and 40,075 AMG were identified from 1981 to 2017 from TTF and Taiwan healthcare public data, respectively. Subjects were further grouped by their graduation year: 1981–1994, 1995–2001, 2002–2011, and after 2012. Ranges were based on implementation dates of new health care policies.

**Results:**

The percentages of female AMG increased from 10.9% before 1994 to 32.6% after 2012 (linear trend, *P* < 0.001). Similarly, the percentages of female TMG increased from 23.1% before 1994 to 42.4% after 2012 (linear trend, *P* = 0.003). In contrast to 2% of AMG, the percentages of TMG who became dermatologists increased from 11% to 20.5% (linear trend, *P* = 0.024). TMG favored dermatology, ophthalmology, and neurology, and avoided general surgery (*P* < 0.001). While still higher than AMG, the percentages of TMG working in medical centers dropped significantly from 58% during 1981–1994 to 33.3% during 1995–2001 (*P* = 0.035). This coincided with the launch of National Health Insurance in 1995. Finally, though more than half of TMG previously worked in Northern Taiwan, they have recently moved to Central Taiwan.

**Conclusions:**

The percentages of female AMG and TMG reached 32.6% and 42.4%, respectively, after 2012. TMG prefer to choose dermatology, ophthalmology, and neurology, but avoid general surgery. Changes in health policy, reimbursement policy, and medical education may be associated with AMG and TMG career choices.

## Background

The Tsungming Tu Foundation (TTF) was founded to honor Doctor Tsungming Tu, who was born in Tamsui, Formosa (Taiwan) in 1893, and was the first Taiwanese MD/PhD graduate from Kyoto University in 1922. He became the first Taiwanese professor in Japan’s pre-1945 imperial university system at Taihoku Imperial University (established in 1928, now National Taiwan University). A pioneer in Taiwan medical education, he founded Kaohsiung Medical College, the first private medical school in Taiwan (now Kaohsiung Medical University) where he became the first University President in 1954.

The TTF chooses recipients for the Tsungming Tu Award, which is divided into three categories: (A) The Commemorative Speech Award is awarded to those recommended at the annual meeting of the Taiwan Medical Association (TMA), the official physician organization in Taiwan since 1902. (B) The Excellent Graduation Award is awarded to top medical school graduates, one per year from each medical school in Taiwan since 1981. (C) The Excellent Paper Award is awarded to the author of an outstanding original research paper published in the Journal of Formosa Medical Association (JFMA, also established in 1902), the official journal of TMA.

The second category, specifically, has provided awards to each top medical graduate (TMG) from each medical school in Taiwan (currently 10 medical schools) every year since 1981. However, the trends of gender distribution among TMG and all medical graduates (AMG) in the past three decades have not been officially reported. Furthermore, how TMG and AMG differ from one another in specialty choice is unknown, including differences in working in primary care versus medical centers, or differences in the geographic regions for practicing.

In the past, there was a significant disparity in medical professions between males and females. Females were excluded from medical education until the nineteenth century, when women started to gain access to medical universities. For example, the first medical school for women in the USA, the Woman’s Medical College of Pennsylvania, was built in Philadelphia in 1850. Similarly, gender disparities were also historically present in the field of dermatology [1]. We became interested in medical graduate gender disparity after one report from the Organization for Economic Co-operation and Development (OECD) nations showed that the percentage of female physicians varied among countries; the highest was 70%, and the lowest was 18% in Estonia and Japan, respectively [2]. Recent reports of discriminative gender policies in medical school admission exams at several medical schools in Japan further highlighted the importance of investigating gender discrimination in Japan [3]. In Taiwan, systems for health care and public health were mostly adopted from the Japanese during Japanese colonization. Similar to Japan, females only comprised 10–15% of medical students in Taiwan in 1980. Therefore, it is interesting to measure the career choices of medical graduates by gender.

Several important health policies issued by Taiwan health authorities may have had dramatic impacts on health service providers and the career planning for medical graduates. For example, National Health Insurance (NHI), covering 99% of Taiwanese residents, was established in 1995 to provide affordable and quality health care services with global budget control but trimmed physician fees. This may have significantly affected work life, home life, working hours, and income for medical and paramedical workers. Another example was the annual cap on specialty residency training capacity launched in 2001 to promote balanced distributions of physician resources among various specialties. This cap included dermatology, ophthalmology, rehabilitation, and several other minor specialties. Understanding how these health policies impacted the career development of TMG and AMG requires further investigation. Herein, we aimed to study whether TMG differ from AMG in gender, specialty, affiliated institutions, and geographic regions in the last 30 years.

## Methods

A total of 322 TMG were identified from the public website of TTF (http://www.tutsungming.org.tw/). Data on the year, gender, specialty, primary care/centers, and geographic regions in Taiwan of 40,075 AMG from 1981 to 2017 were acquired from the public data of the Division of Statistics of Ministry of Education and Taiwan Medical Association (Table 1). Based on the two health policies mentioned previously and an average 7-year gap for TMG to get board approval after graduation, we further divided the 322 TMG into four groups by their graduation year. These subgroups were 1981–1994, 1995–2001, 2002–2011, and after 2012. The specialty data from 2016 to 2017 were excluded when calculating the percentage of dermatologists during 2012–2017 because they were unavailable.

**Table 1:** Proportions of genders and dermatologists among top medical graduates and all medical graduates from 1981 to 2017

	N of awarded top medical graduates (TMG)					N of all medical graduates (AMG)				
Year	M	F	F%	Total	Dermatologist	M	F	F%	Total	Dermatologist
1981–1994	70	21	23.1	91	10 (11.0%)	12,240	1503	10.9	13,743	339 (2.5%)
1995–2001	39	24	38.1	63	13 (20.6%)	5616	1542	21.5	7158	218 (3.0%)
2002–2011	56	46	45.1	102	26 (25.5%)	8554	3174	27.1	11,728	315 (2.7%)
2012–2017	38	28	42.4	66	12 (20.5%)^a^	5021	2425	32.6	7446	83 (1.7%)^a^
Total	203	119		322	61 (18.9%)	31,431	8644		40,075	955 (2.4%)

*F* female, *M* male, *N* numbers

^a^The percentage of dermatologists was calculated by excluding data from 2016 to 2017

SPSS software (IBM SPSS Statistics for Windows, Version 22.0. Armonk, NY: IBM Corp.) was used to perform statistical analyses. One-way analysis of variance (ANOVA) and post-hoc tests were applied to evaluate the differences in gender distribution, different specialties, practice organization, and geographic location between the TMG and AMG. We also conducted Pearson’s Chi-squared test to analyze the differences in categorical variables of the choice of medical disciplines. Statistical significance was defined as a *P*-value < 0.05, with a two-tailed test.

## Results

### Gender trend among TMG and AMG during the last three decades

**Fig. 1:**
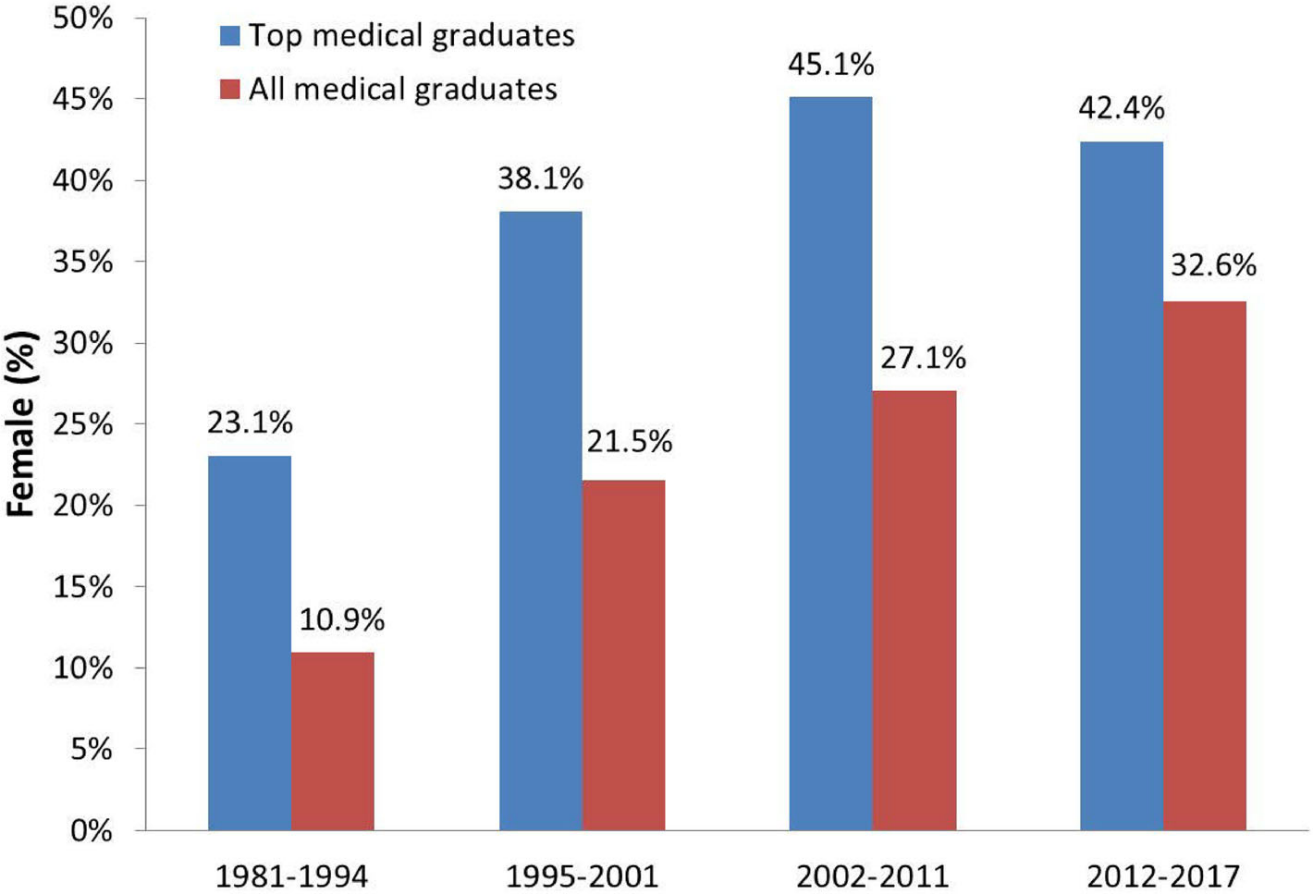
The percentages of females among TMG and AMG since 1981 in Taiwan. The percentages of female medical graduates among TMG and AMG were significantly increased from 1981 to 2017 (linear trend, *P* = 0.003 and *P* < 0.001, respectively)

There were 31,431 male graduates and 8644 female graduates during the 30 years measured (M: F = 3.64: 1). During the past three decades, the percentage of all female graduates increased significantly: 10.9% during 1981–1994, 21.5% during 1995–2001, 27.1% during 2002–2011, and 32.6% during 2012–2017 (linear trend, *P* < 0.001) (Fig. 1). In contrast, among the 322 TMG, there were 203 males and 119 females (M: F = 1.7: 1). The percentage of female TMG also increased significantly: 23.1% during 1981–1994, 38.1% during 1995–2001, 45.1% during 2002–2011, and 42.4% after 2012 (linear trend, *P* = 0.003). These results showed a tendency of increasing proportions of females choosing to be medical doctors in the last three decades. Interestingly, although the female doctors encompass only~ 30% of AMG, they comprise more than 40% of TMG.

### TMG in dermatology

**Fig. 2:**
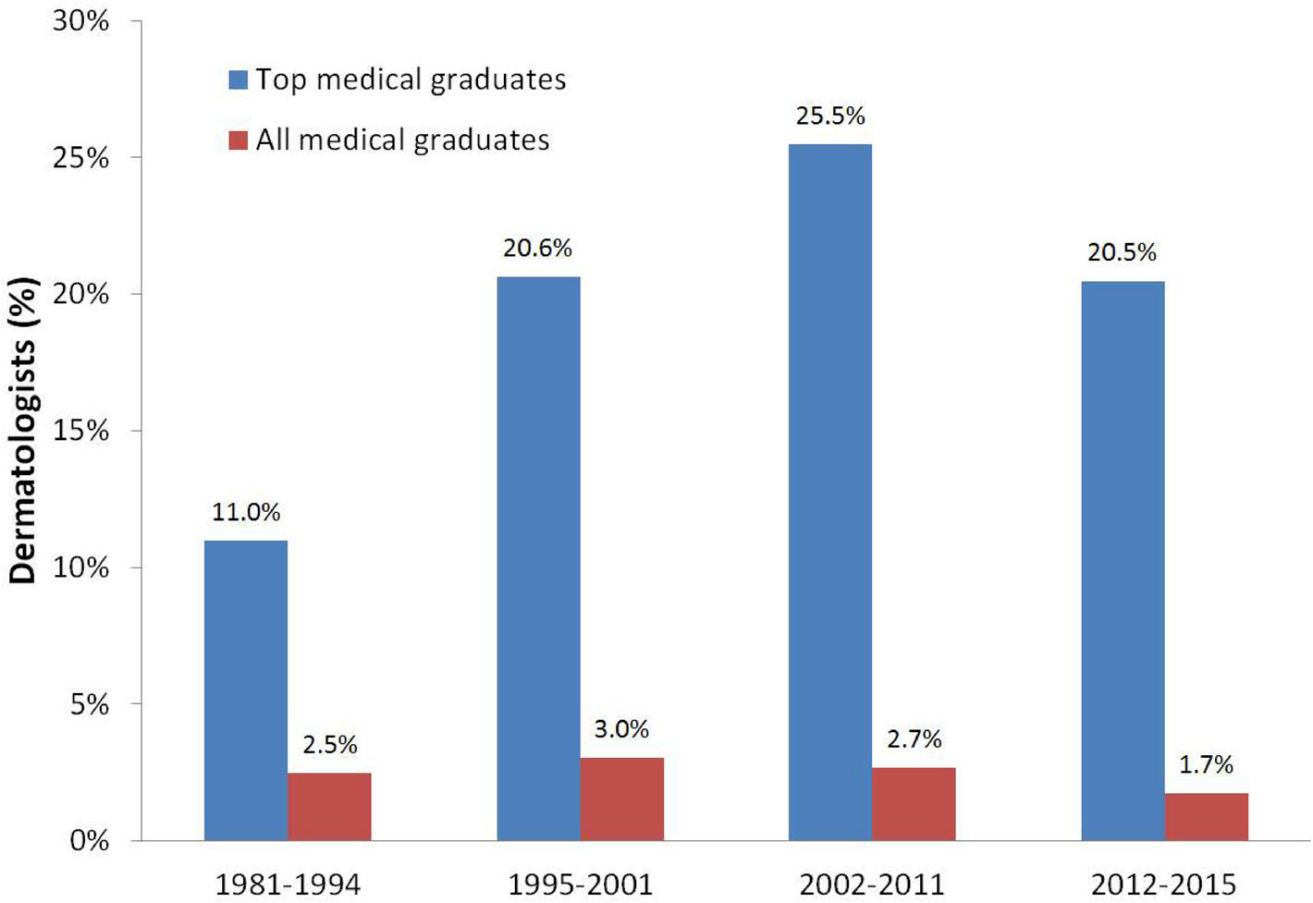
The percentages of dermatologists among TMG and AMG since 1981 in Taiwan. The percentages of those who chose dermatology among TMG increased significantly (linear trend, *P* = 0.024). However, the percentage of dermatologists among AMG has remained consistent since 1981 (linear trend, *P* = 0.111)

During the last three decades, consistently 2–3% of AMG chose dermatology as their specialty (linear trend, *P* = 0.111). In contrast, of the 322 TMG, 61 chose dermatology as their career choice (18.9%). The percentages of TMG choosing dermatology were 11% during 1981–1994, 20.6% during 1995–2001, 25.5% during 2002–2011, and 20.5% during 2012–2015 (linear trend, *P* = 0.024) (Fig. 2).

### Career choices

**Fig. 3:**
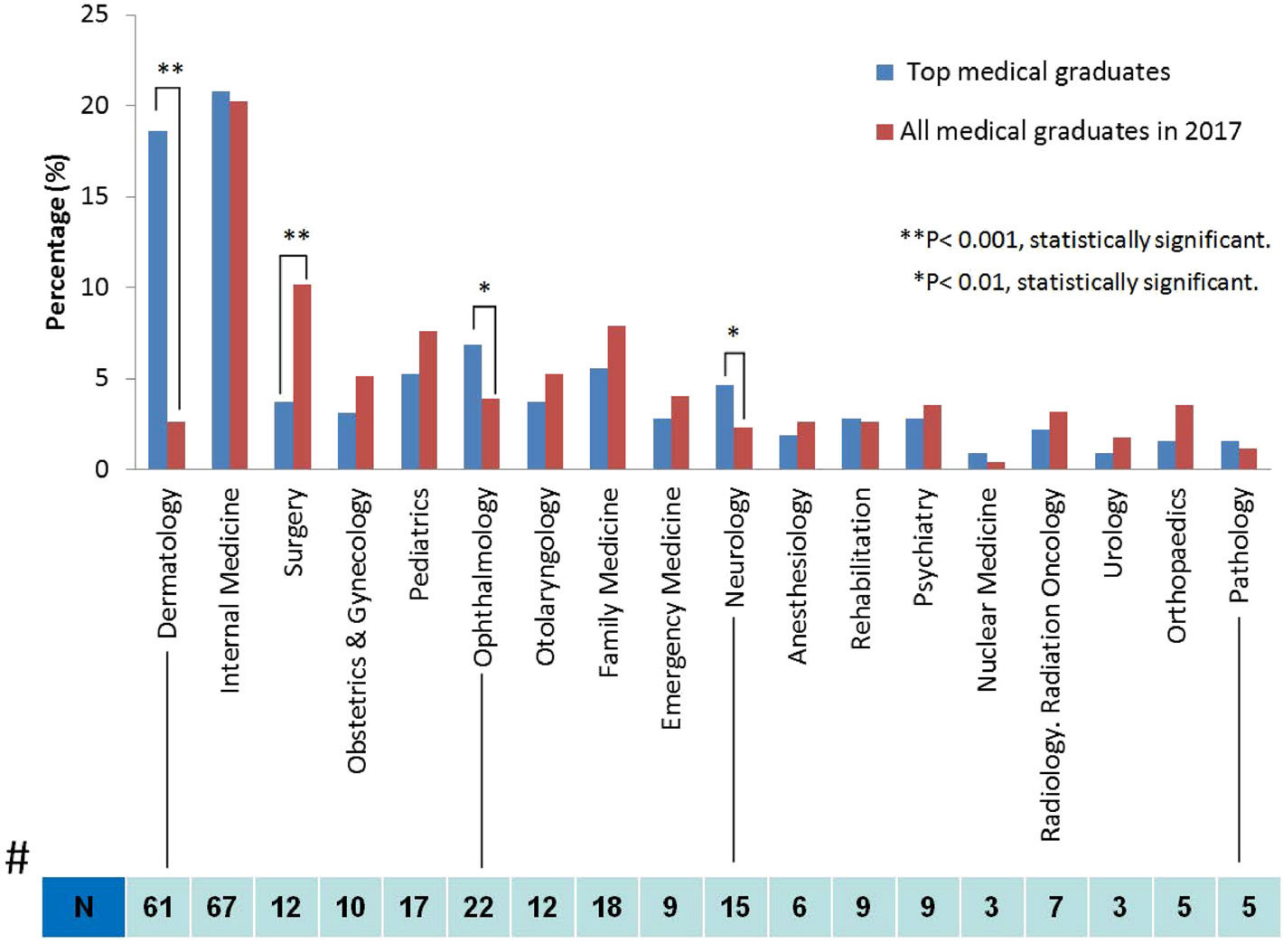
The choices of medical disciplines among top and all medical graduates in 2017 in Taiwan. The data showed significantly increased percentages of TMG practicing dermatology (*P* < 0.001), ophthalmology (*P* < 0.01), and neurology (*P* < 0.01). In contrast, fewer TMG chose the surgery (*P* < 0.001) as their specialty. N reflects the absolute numbers of TMG in each specialty. Note: missing data (*N* = 17), working abroad (*N* = 9), receiving post-graduate training (*N* = 3), working at an aesthetic clinic (*N* = 1), studying public health (*N* = 1), death (*N* = 1)

To investigate the trends of medical disciplines chosen by TMG, we compared TMG with AMG in terms of specialty choice in 2017. The result showed that the percentage who chose dermatology was 18.9% among top graduates versus 2.6% among all graduates (*P* < 0.001). Similar results were obtained for ophthalmology (6.8% vs. 3.9%, *P* < 0.01) and neurology (4.7% vs. 2.3%, *P* < 0.01). In contrast, fewer percentages of TMG chose general surgery compared with AMG (3.7% vs. 10.2%, *P* < 0.001) (Fig. 3). Overall, data missing for TMG were 5.3% (*N* = 17), and a small portion of graduates eventually decided to work in other countries (*N* = 9, percentage = 2.8%). To address whether the differences in specialty choice in the TMG simply a function of the fact that there is a higher proportion of women than men in the TMG, we stratified TMG by gender, by year, and by four specialties (dermatology, ophthalmology, neurology, and surgery), that showed significances initially (Table 2). The percentages of female TMG choosing dermatology were 50% during 1981–1994, 30.8% during 1995–2001, 34.6% during 2002–2011, and 58.3% during 2012–2017. It showed variable results with no significant trend. Similarly, the percentages of female TMG choosing ophthalmology, neurology, and surgery during these years did not change significantly.

**Table 2:** Proportions of genders among top medical graduates (TMG) with specific specialties from 1981 to 2017

	Dermatology				Ophthalmology				Neurology				Surgery			
Year	M	F	F%	Total	M	F	F%	Total	M	F	F%	Total	M	F	F%	Total
1981–1994	5	5	50	10	4	3	42.9	7	3	0	0	3	6	0	0	6
1995–2001	9	4	30.8	13	5	3	37.5	8	0	1	100	1	2	1	33.3	3
2002–2011	17	9	34.6	26	0	1	100	1	2	6	75	8	1	0	0	1
2012–2017	5	7	58.3	12	2	4	66.7	6	2	1	33.3	3	1	1	50	2
Total	36	25		61	11	11		22	7	8		15	10	2		12

*F%* F/Total of each medical specialty, *F* female, *M* male

### Primary, secondary, or tertiary health care

**Fig. 4:**
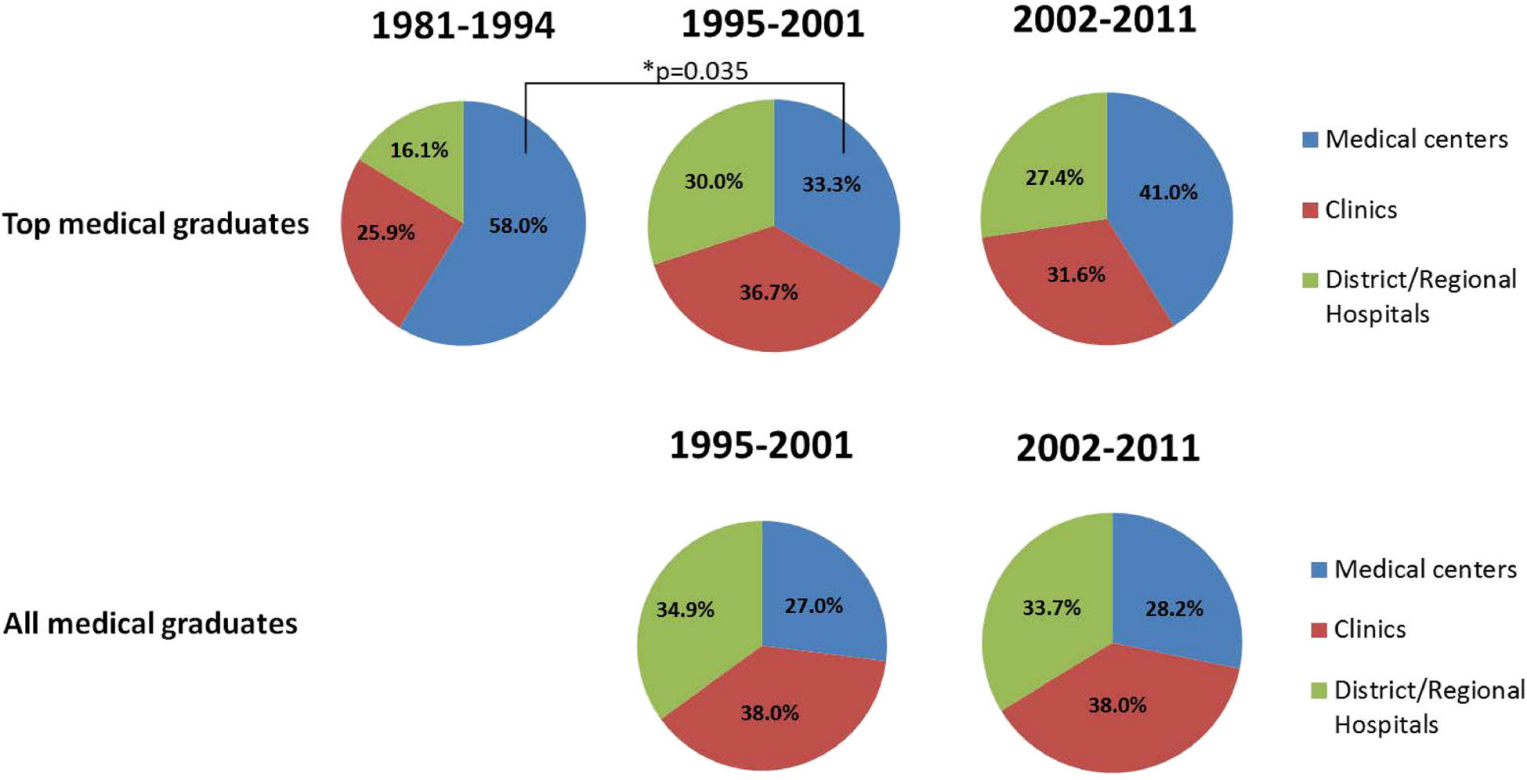
Practice organization among TMG and AMG by graduation year. The data showed that TMG tended to work in medical centers more often than AMG did in the same year interval from 1995 to 2011. The percentages of TMG in medical centers dropped significantly: 58% during 1981–1994 to 33.3% during 1995–2001 (*P* = 0.035), but rebounded slightly to 41% during 2002–2011. The data of AMG during 1981–1994 were unavailable

To investigate whether TMG differed from AMG in choosing to work in primary care clinics or to stay in medical centers, we grouped the affiliated institution into three categories: medical centers, private clinics, and district/regional hospitals based on different levels of health care providers (Fig. 4). The medical centers are defined as hospitals providing tertiary care (also called tertiary referral centers or tertiary referral hospitals). However, the private clinics and district/regional hospitals are hospitals that provide primary and secondary care, respectively. Based on the year of graduation, TMG and AMG were categorized into 1981–1994, 1995–2001, and 2002–2011. The students who graduated after 2012 were excluded from this study because most of them remained in the medical residency training programs. We found that more TMG worked in medical centers compared with AMG in the same period. However, the percentages of TMG working in medical centers dropped by almost half, with 58% during 1981–1994 to 33.3% during 1995–2001 (*P* = 0.035). This may be associated with the launch of NHI in 1995, which provides more affordable medical care to patients at the expense of increasing the workload of medical teams. This drop rebounded slightly to 41% during 2002–2011, which may have been related to the establishment of an annual cap on residency capacity in some minor specialties in 2001.

### Location of practice

**Fig. 5:**
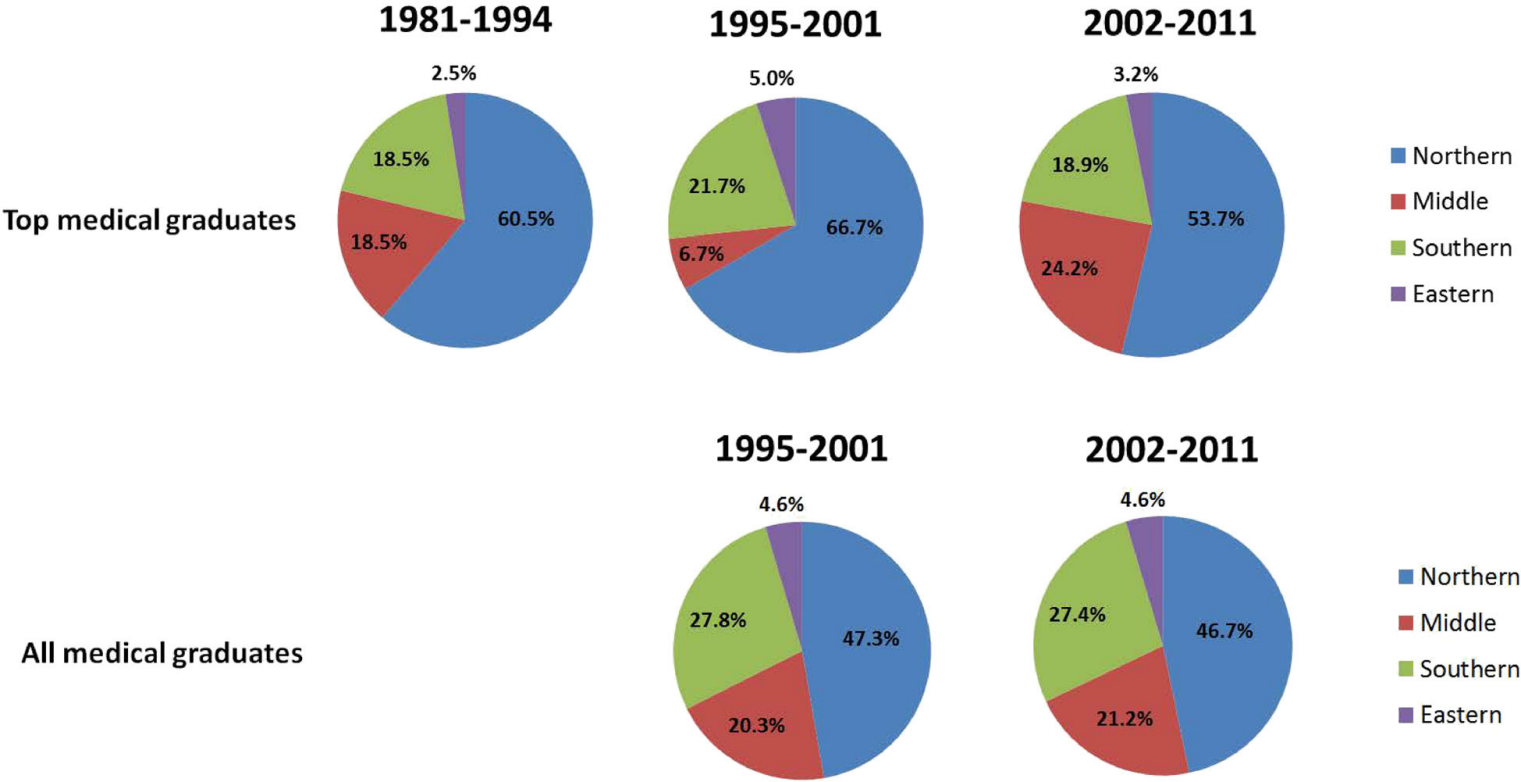
Practice geographic location among TMG and AMG by graduation year. The data showed that more than half of TMG worked in Northern Taiwan from 1981 to 2011. There was a shift from northern to middle regions from 1995–2001 to 2002–2011 for TMG. The data of AMG during 1981–1994 were unavailable. The data of Eastern Taiwan also included offshore islands of Taiwan

For administrative purposes, the geographic districts of Taiwan were divided into four main regions, including Northern (the most developed region, including Taipei), Middle, Southern (the next developed region, including Kaohsiung, the largest harbor), and Eastern (the least developed region in mountainous areas). For health administrative reasons, offshore islands of Taiwan were grouped into the Eastern district (the rural region). We further categorized graduation years into 1981–1994, 1995–2001, and 2002–2011, and compared the differences between AMG and TMG (Fig. 5). Medical graduates after 2012 were excluded from this study because most of them remained in the medical residency training programs. The data showed that more than half of TMG worked in Northern Taiwan from 1981 to 2011, though the percentage decreased after 2002. TMG who worked in Northern Taiwan dropped from 66.7% in 1995–2001 to 53.7% in 2002–2011 with a shift from the northern region to the middle region from 1995–2001 to 2002–2011.

## Discussion

We showed the percentages of females among AMG increased significantly from 10.9% to 32.6% during 1981–2017 because the rising awareness of gender equality in Taiwan. On the other hand, in Japan, Yamazaki et al. designed open-ended questionnaires and mailed them to the female Japanese physicians. The study found that the main challenges facing Japanese female doctors were from Japanese society, family responsibilities, and work environment. They also reported gender inequities and sexual harassment in the workplace [4]. In contrast to Japan, the other OECD member countries of Scandinavia, such as Norway, Denmark, Sweden, and Finland, have higher percentages of female physicians ranging from 42% to 56%. These circumstances are associated with progressive work-life policies and changes of work-life balance. Healthy work environments that are female-friendly would be more likely to attract younger or female physicians to those less-chosen specialties [5]. A study from Switzerland with 5038 residents showed that work and time-related issues were important to female residents. In addition, female residents considered career-related aspects to be less important than male residents did [6]. Nonetheless, physicians in both gender take lifestyles into consideration when selecting their specialty [7].

Physician gender impacts the healthcare of underserved communities, the promotion of medical research, and tending to patient needs. In US, Gordon et al. found the proportion increased significantly among female residents in all medical specialties through 36 years. For dermatology, there was a 1.1% increment increase in female residents per year (*P* < 0.001) [8]. In UK, Barat et al. found the dermatology specialty is popular among female graduates [9]. Although the female TMG increased significantly from 1981 to 2017 in our study, the percentages of female TMG who became dermatologists were similar.

In the 1980’s, medical students considered lifestyle to be the least important when choosing a medical specialty [10]. However, there has been a substantial change in preferences in recent decades. Sometimes, the word “ROAD” is used to depict the specialties that exhibit a great lifestyle—radiology, ophthalmology, anesthesia, and dermatology [11, 12]. On the other hand, career choices for general practice remain low [13]. These changes also happened in US, for new generation of medical students. Regardless of genders, medical graduates need more flexibility and protected time for friends and family during their careers [14]. In 1990, Schwartz et al. further classified medical specialties into controllable lifestyles and uncontrollable lifestyles. The specialties of controllable lifestyles are dermatology, ophthalmology, neurology, radiology, emergency medicine, anesthesiology, pathology, psychiatry, and otolaryngology, while other specialties, such as general surgery, internal medicine, obstetrics/gynecology, pediatrics, family practice, orthopedics, and urology, were sorted as uncontrollable lifestyles [15]. A study that analyzed the specialty choices of medical students in the US between 1990 and 2003 revealed that both males and females tended to choose specialties with controllable lifestyles [16]. This result is also compatible with the “ROAD” as mentioned above. We found that TMG tend to choose dermatology, ophthalmology, and neurology as their careers, and surgery specialty was the least chosen.

Janet et al. highlighted the impacts of role models and mentors for medical students and residents when they are setting career goals [17]. Previous studies in Canada and US have shown that experiencing sex-based discrimination and a lack of female role models discourages female medical students from choosing general surgery as their careers [18, 19]. In addition, lifestyle and workload issues also have a great influence on both male and female surgeons’ career plans [7]. However, it should be noticed that junior doctors’ early career choices may not predict their career destination based on a UK study [20]. Although the numbers of female medical doctors have increased, inequalities of leadership and prestige between males and females still do exist in academic dermatology. Based on one study from the American College of Mohs Surgery Membership in 2009, the percentage of female residency program directors was 28%, and the percentage of female department chairs was only 16%. In 2016, the percentage of female dermatology residency program directors increased greatly to 47.9%. However, the proportion of chairpersons was still relatively lower (23.5%) [21].

A previous survey in the United States showed that about 11–16% of dermatology residents would work in an academic department after their residency training. To investigate the trend of dermatologists’ workplaces, Resneck et al. analyzed the data of 1425 dermatologists, showing that the percentage of academic dermatologists was only 6% [22]. Dermatologists who work in medical centers might need to spend more time at work, such as doing research, teaching students, and writing papers. Fewer medical students choose to stay in medical centers, and this may be attributed to unique workforce challenges in academic dermatology. The above phenomenon may be related to the significant decrease of the percentage of TMG working in medical centers in Taiwan, from 58% during 1981–1994 to 33.3% during 1995–2001.

Since the launch of National Health Insurance in 1995, it has provided affordable and quality health care services with global budget control but trimmed physician fees. This health policy may affect work life, home life, working hours, and income for medical and paramedical workers significantly. As shown in Fig. 4, we found the percentage of TMG working in medical centers dropped significantly from 58% during 1981–1994 to 33.3% during 1995–2001 (*P* = 0.035). The changes may be associated with the introduction of the National Health Insurance.

### Limitations

Because this is an observational study, the causative association could not be well ascertained. This is a study within a unique health care claim system in Taiwan and may not be extrapolated to other countries.

## Conclusions

Over the past few decades, an increased focus on the impact of National Health Insurance in Taiwan and the annual cap of dermatology residency capacity may have an influence on the specialty choices of TMG. The proportion of female TMG has grown over the last three decades. These TMG tended to choose the specialties of dermatology, ophthalmology, and neurology, but avoided general surgery during the past three decades. The changes in health policy and reimbursement policy may have been associated with the career choices among AMG and TMG. To the best of our knowledge, this is the first study investigating the career choices of top medical students’ medical specialties, with a specific focus on gender, over 30 years in Taiwan.

## Data Availability

The datasets used and/or analyzed during the current study are available from the corresponding author on reasonable request. The list of TMG from Tsungming Tu Foundation from 1981 to 2019 is available in the public website of http://www.tutsungming.org.tw/award.htm.

## Abbreviations

AAD: American Academy of Dermatology
ANOVA: Analysis of variance
JFMA: Journal of Formosa Medical Association
NHI: National Health Insurance
OECD: Organization for Economic Co-operation and Development
AMG: All medical graduates
ROAD: Radiology, ophthalmology, anesthesia, dermatology
TMA: Taiwan Medical Association
TMG: Top medical graduates
TTF: Tsungming Tu Foundation

## References

[1] Franca K, Ledon J, Savas J, Nouri K (2014). Women in medicine and dermatology: history and advances. An Bras Dermatol.

[2] Ramakrishnan A, Sambuco D, Jagsi R (2014). Women’s participation in the medical profession: insights from experiences in Japan, Scandinavia, Russia, and Eastern Europe. J Women’s Health (Larchmt).

[3] McCurry J (2018). Tokyo medical school admits changing results to exclude women.

[4] Yamazaki Y, Kozono Y, Mori R, Marui E (2011). Difficulties facing physician mothers in Japan. Tohoku J Exp Med.

[5] Koike S, Matsumoto S, Kodama T, Ide H, Yasunaga H, Imamura T (2009). Estimation of physician supply by specialty and the distribution impact of increasing female physicians in Japan. BMC Health Serv Res.

[6] van der Horst K, Siegrist M, Orlow P, Giger M (2010). Residents’ reasons for specialty choice: influence of gender, time, patient and career. Med Educ.

[7] Riska E (2011). Gender and medical careers. Maturitas..

[8] Bae G, Qiu M, Reese E, Nambudiri V, Huang S (2016). Changes in sex and ethnic diversity in dermatology residents over multiple decades. JAMA Dermatol.

[9] Barat A, Goldacre MJ, Lambert TW (2018). Career choices and career progression of junior doctors in dermatology: surveys of UK medical graduates. Dermatol Res Pract.

[10] Tardiff K, Cella D, Seiferth C, Perry S (1986). Selection and change of specialties by medical school graduates. J Med Educ.

[11] DeZee KJ, Byars LA, Magee CD, Rickards G, Durning SJ, Maurer D (2013). The R.O.A.D. confirmed: ratings of specialties’ lifestyles by fourth-year US medical students with a military service obligation. Fam Med.

[12] Lambert TW, Goldacre MJ, Bron AJ (2008). Career choices for ophthalmology made by newly qualified doctors in the United Kingdom, 1974-2005. BMC Ophthalmol.

[13] Lambert TW, Smith F, Goldacre MJ (2018). Career specialty choices of UK medical graduates of 2015 compared with earlier cohorts: questionnaire surveys. Postgrad Med J.

[14] Sanfey HA, Saalwachter-Schulman AR, Nyhof-Young JM, Eidelson B, Mann BD (2006). Influences on medical student career choice: gender or generation?. Arch Surg.

[15] Schwartz RW, Haley JV, Williams C, Jarecky RK, Strodel WE, Young B (1990). The controllable lifestyle factor and students’ attitudes about specialty selection. Acad Med.

[16] Lambert EM, Holmboe ES (2005). The relationship between specialty choice and gender of U.S. medical students, 1990-2003. Acad Med.

[17] Bickel J (2001). Gender equity in undergraduate medical education: a status report. J Womens Health Gend Based Med.

[18] Park J, Minor S, Taylor RA, Vikis E, Poenaru D (2005). Why are women deterred from general surgery training?. Am J Surg.

[19] Gargiulo DA, Hyman NH, Hebert JC (2006). Women in surgery: do we really understand the deterrents?. Arch Surg.

[20] Barat A, Goldacre MJ, Lambert TW (2019). Junior doctors’ early career choices do not predict career destination in neurology: 40 years of surveys of UK medical graduates. BMC Med Educ.

[21] Shi CR, Olbricht S, Vleugels RA, Nambudiri VE (2017). Sex and leadership in academic dermatology: a nationwide survey. J Am Acad Dermatol.

[22] Resneck JS, Tierney EP, Kimball AB (2006). Challenges facing academic dermatology: survey data on the faculty workforce. J Am Acad Dermatol.

